# The role of plasma inflammatory markers in late-life depression and conversion to dementia: a 3-year follow-up study

**DOI:** 10.1038/s41380-025-02908-2

**Published:** 2025-02-08

**Authors:** M. Bocharova, T. Borza, L. O. Watne, K. Engedal, J. T. O’Brien, G. Selbæk, A. V. Idland, J. Hodsoll, A. H. Young, D. Aarsland

**Affiliations:** 1 Department of Psychological Medicine, Institute of Psychiatry, Psychology and Neuroscience, King’s College London, London, UK; 2 Research Centre for Age-Related Functional Decline and Disease, Innlandet Hospital Trust, Ottestad, Norway; 3 Department of Geriatric Medicine, Akershus University Hospital, Lørenskog, Norway; 4 Institute of Clinical Medicine, Campus Ahus, University of Oslo, Oslo, Norway; 5 Norwegian National Centre for Ageing and Health, Vestfold Hospital Trust, Tønsberg, Norway; 6 Department of Psychiatry, University of Cambridge School of Clinical Medicine, Cambridge, UK; 7 Department of Anesthesiology, Akershus University Hospital, Lørenskog, Norway; 8 Department of Biostatistics & Health Informatics, Institute of Psychiatry, Psychology and Neuroscience, King’s College London, London, UK; 9 Division of Psychiatry, Imperial College London, London, UK; 10 Centre for Age-Related Diseases, Stavanger University Hospital, Stavanger, Norway

**Keywords:** Prognostic markers, Depression

## Abstract

Late-life depression (LLD) has been linked to increased likelihood of dementia, although mechanisms responsible for this association remain largely unknown. One feature frequently observed in both LLD and dementia is elevated levels of plasma inflammatory markers. The present study aimed to compare the levels of 12 plasma inflammatory markers between older people with LLD and controls, and to explore whether these markers, along with clinical characteristics, can predict dementia in patients with LLD within 3 years of follow-up. Using multiple linear regression with stepwise adjustment, we compared levels of plasma inflammatory markers (IL-1β, IL-1ra, IL-6, IL-10, IL-17a, IL-18, IL-33, TNFα, CD40L, IFN-γ, CCL-2 and CCL-4) between 136 inpatients with LLD (PRODE cohort) and 103 cognitively healthy non-depressed controls (COGNORM cohort). In the PRODE cohort, follow-up data was available for 139 patients (of them 123 had data on baseline plasma inflammatory markers); 36 (25.9%) developed dementia by Year 3 (n = 31 for those with cytokine data). Using Cox proportional hazards regression, we explored whether inflammatory markers and clinical characteristics of LLD (age of onset, treatment response, number of episodes) predicted progression to dementia during follow-up. Levels of IL-1ra, CCL-2, CCL-4, IFN-γ and IL-17a were significantly higher in LLD patients compared to controls in the majority of models. However, none of the inflammatory markers predicted progression from LLD to dementia in the PRODE cohort. Among clinical features, only poor response to treatment significantly predicted higher risk of progression to dementia.

## Introduction

Late-life depression (LLD) – a term describing a depressive episode or “clinically significant depressive symptoms” occurring in people aged 60 or above - is a serious medical condition which affects, according to various estimates and definitions, from 2.6–35.1% of older adults, and is associated with substantial healthcare burden [1, 2]. The importance of elucidating the biological underpinnings of LLD consists in that fact that, besides being a common and burdensome condition itself, LLD is associated with increased risk of developing dementia later in life [3, 4], as well as with accelerated cognitive decline [5]. The latest Lancet commission report on dementia prevention reaffirmed depression as an important modifiable risk factor for dementia, also highlighting that treating depression has a potential to reduce this risk [6].

One of the potential mechanisms linking LLD and dementia is neuroinflammation. The main immunomodulating agents researched in this respect are cytokines and chemokines– small proteins that act as signalling molecules and regulate the immune response. Elevated levels of systemic and neuro-inflammation in depression have been shown to negatively affect neurogenesis, leading to neurodegeneration and reduced hippocampal volumes [7, 8].

Meta-analyses have shown elevated levels of pro- and anti-inflammatory cytokines and chemokines in adult major depression (aMDD), including IL-6, CRP, TNF-α, IL-1ra, IL-1β, IFN-γ, IL-10, IL-18 and a few other [9, 10]. However, less is known about inflammation specifically in LLD. A meta-analysis by Ng et al. focused on peripheral levels of Interleukin-1 beta (IL-1β), Interleukin-6 (IL-6), Tumor Necrosis Factor-α (TNF- α) and C-Reactive Protein (CRP) and demonstrated elevated levels of IL-1β and IL-6 in LLD compared to controls, but not of TNF- α or CRP [11]. A few other studies have reported an association between LLD and elevated levels of pro-inflammatory cytokines [12–14].

Dementia, and specifically Alzheimer’s disease, is also characterized by increased release of pro-inflammatory cytokines, driven by microglia overactivation and triggering neuronal damage and synaptic dysfunction [15, 16]. Meta-analyses confirm elevated levels of peripheral and CSF inflammatory markers in dementia [11, 17]. However, very few studies have directly addressed the interplay between LLD, inflammatory markers and risk of dementia or cognitive decline.

The present study examined the role of an array of plasma inflammatory markers in LLD and conversion to dementia over 3 years of follow-up using a naturalistic prospective cohort of older people seeking hospital treatment for LLD (PRODE) and a control cohort of non-depressed cognitively stable older people (COGNORM). We hypothesized that LLD would be characterized by elevated levels of plasma inflammatory markers compared to non-depressed controls, and plasma inflammatory markers in LLD could predict cognitive decline and/or progression to dementia during 3 years of follow-up. Additionally, we explored whether clinical characteristics of LLD, such as age of onset; treatment response and number of previous episodes, could predict progression to dementia.

## Methods

### Sample

Participants with LLD were recruited to the PRODE (PROgnosis of Depression in the Elderly) cohort. PRODE is a prospective naturalistic study of 152 depressed patients aged 60 or above, recruited across several clinical centers in Norway between December 1st, 2009 and January 1st, 2013. Patients were included if they were diagnosed with depression according to ICD-10 and did not have a diagnosis of dementia at enrollment. The majority of patients (n = 146; 96%) were admitted to psychiatric units for depression. All patients received treatment for depression in accordance with standard treatment protocols. Patients were assessed in four waves: on admission, at discharge (median 1.68 months post-admission; min 0.33- max 9.2 months); one year after discharge (median 11.34; min 4.64 – max 18.51 months post-discharge), and again 3 years after discharge (median 36.33 months, min 29.49-max 38.66 post-discharge). Clinical and neuropsychological data were collected by clinicians and trained researchers using a wide range of standardized clinical, psychiatric, and neuropsychological assessment tools as described below. A detailed description of the cohort has been previously published [18].

A separate Norwegian cohort using partially matching measurement instruments, Cognitively Healthy Control Group-study (COGNORM), was recruited from 2012 to 2013 at Oslo University Hospital and Diakonhjemmet Hospital, Oslo. A total of 172 patients, aged 64 years or older in the year of inclusion, undergoing elective gynaecological, orthopaedic, or urological surgery in spinal anaesthesia, were assessed with a multi-domain battery of cognitive tests prior to surgery. Subjects were excluded if they had dementia, previous stroke with sequelae, Parkinson’s disease or other acknowledged or suspected brain diseases likely to influence cognition. Subjects were also excluded if they had a current depression score above 8 on the Montgomery–Åsberg Depression Rating Scale (MADRS). The COGNORM cohort was followed annually after inclusion, with participants exhibiting cognitive decline during follow-up assessments excluded from the study to retain focus on cognitively healthy older adults. The sample was previously described in detail elsewhere [19]. None of the 103 COGNORM participants included in the present study were known to develop dementia by year 3 (see “Missing data and attrition” for details). Due to the absence of dementia cases in the control sample and the exclusion of participants with signs of cognitive decline at each follow-up assessment, the comparison of cognitive outcomes at year 3 between PRODE and COGNORM participants was not feasible. Therefore, this study utilized the COGNORM sample only to perform a cross-sectional analysis of differences in inflammatory biomarkers between the LLD group and controls.

### Procedures and assessment

#### Mental health

The diagnosis of depression in the PRODE cohort was made by a psychiatrist or clinical psychologist in accordance with ICD-10 criteria [20].

The severity of current depressive symptoms was assessed with the Norwegian version of the Montgomery and Aasberg Depression Rating Scale (MADRS; [21, 22]. The scale consists of 10 items assessing depressive symptoms that are rated on a scale from 0 to 6 (total score 0–60); higher scores indicate more severe symptoms.

Information on psychiatric history, including the number of depressive episodes and age at first onset, was gathered from case notes and structured interviews with patients and caregivers. Early-onset depression (EOD) was defined as occurring before age 60, and late-onset depression (LOD) at 60 or later. However, some studies have used age 50 as the threshold [23], so we conducted an additional analysis using this cutoff (See Tables S1 and S2 in Supplement). The number of prior depressive episodes was categorized into three levels: no prior episodes, one episode, and two or more episodes.

#### Cognitive assessment on admission, discharge and Year 1

General cognitive performance was assessed using the Mini Mental State Examination (MMSE) scale, with a score range of 0–30, where lower score indicates worse performance [24].

The neuropsychological test battery included several tests assessing performance on different cognitive domains. Trail Making Test (TMT) measures attention, visual screening ability, and processing speed [25, 26]. Results on the TMT were scored according to existing age-adjusted norms derived from Ivnik et al. [27]. COWAT word and category fluency is a test of verbal fluency measuring spontaneous production of words belonging to the same category or beginning with some designated letter [28]. Immediate recall, delayed recall and recognition were measured using the Consortium to Establish a Registry for Alzheimer’s Disease (CERAD) tests [29, 30].

#### Dementia diagnosis

The diagnosis of dementia was made according to ICD-10 criteria [20]. At year 1, dementia diagnosis was performed during a follow-up in-person clinical assessment. At year 3, cognition assessment comprised of several rating scales and questionnaires administered over the phone: (a) a validated telephone version of the Mini Mental State Examination (MMSE) was performed in all but 18 participants whose health condition precluded the implementation of the test by telephone; for them, a traditional MMSE was administered in the primary health service [31]; (b) the Informant Questionnaire on Cognitive Decline in the Elderly (IQCODE), which contains 16 questions on cognitive changes over time (for the purposes of this study, the period over the last two years was addressed; [32]); the Clinical Dementia Rating scale was used to evaluate cognition and functioning including memory, orientation, judgement and problem solving, community affairs, home and hobbies, and personal care [33]. Based on available information, three researchers, all psychiatrists with a PHD, classified cognition at follow-up into three categories: no cognitive impairment, mild cognitive impairment according to the Winblad criteria [34], and dementia according to ICD-10 [20].

#### Assessment of improvement after treatment (For PRODE cohort)

Improvement of depressive symptoms after treatment was measured based on MADRS scores: (a) as the absolute difference in MADRS score at baseline and post-treatment, where lower scores indicated poorer response to treatment; (b) as “response” defined as at least a 50% improvement in MADRS score, and (c) as “remission” defined as MADRS score below 9 post-treatment. In addition, the CGI-C (clinical global impression - change) scale was used as measure of improvement after treatment [35]. The scale has seven categories, from “much better” to “worse”, and for convenience was dichotomized into patients who improved (n = 114) and patients who did not improve or got worse (n = 37).

### Covariates

Registered demographic characteristics of participants included age, gender, education level (in years), body mass index (BMI; this data was only available for the PRODE cohort) and marital status. All participants in both cohorts were of Scandinavian ethnicity. In addition, smoking status was assessed since cigarette smoke has been previously linked to increased levels of inflammation [36]. Smoking was initially assessed as three categories: “Never smoked”, “Quit smoking in the past” and “Current smoker”, however, exploratory analyses showed no difference in inflammation between participants who had never smoked and those who quit, therefore smoking was recoded as a binary variable.

A recent study of inflammatory markers in LLD argued that the elevation of inflammatory markers in LLD may be attributed to comorbid physical illness and not depression itself [37]; to avoid bias associated with the presence of physical illness, we present results adjusted for comorbid medical diagnoses. Data on comorbid physical ICD-10 diagnoses was available for all participants. Physical comorbidity was coded as binary variables using the following categories: “heart disease” (including diagnoses related to coronary heart disease, heart failure and arrhythmia), hypertension, diabetes, cancer, cerebrovascular disorders, head injuries, and autoimmune disorders. In addition, the score on the General Medical Health Rating (GHMR), a rapid one-item global rating scale of medical comorbidity in dementia patients (four categories: poor, fair, good, very good), was used as a covariate in some of the analyses. GMHR has been found to be a highly reliable “bedsite” rating scale [38]. For the purpose of the analysis, GMHR score was dichotomized to reflect “good” and “poor” health.

### Cytokine analysis

Blood samples were drawn by venepuncture and collected for assessment in sterile serum tubes within 2 weeks of first contact with the patient. Frozen specimen were further shipped to the Oslo University Biobank and stored at −70–80 °C. A total of 12 inflammatory markers were selected based on their previously reported association with depression or cognitive decline/dementia. These included IL-1β, interleukin-1 receptor antagonist (IL-1ra), IL-6, IL-10, IL-17a, IL-18, IL-33, TNF-α, cluster of differentiation 40 ligand (CD40L), interferon (IFN)-γ, and chemokine ligands CCL-2 and CCL-4.

The markers were analyzed according to the manufacturer’s protocol using a bead-based multiplex immunoassay panel (Bio-Techne, Minneapolis, MN) based on xMAP Technology (Luminex, Austin, TX). Inflammatory marker concentrations were calculated using a 5-parameter standard curve based on standards that were supplied by the manufacturer. All analyses were run at the same time, with the exception of 1 of the 10 plates which had to be reanalysed due to a technical error (reanalysis data was used for CD40L and IL-10). Undetected values and values below the lower recommended limit of quantification (LLOQ) were set to 25% of LLOQ, and standard curves with a high LLOQ were selected to obtain a similar sensitivity for all samples analysed. Frequencies of undetected/below-LLOQ values for each of the cytokines are presented in Table S3 in Supplement. All values were reported in picogram/millilitre.

### Statistical analysis

#### Descriptive statistics

Outcome variable normality (plasma cyto- and chemokine levels) was checked using histograms and Q-Q plots. Extreme cytokine values were winsorized to reduce bias while preserving high values, with differences before and after winsorization shown in Table S3 in Supplement. Baseline differences in clinical and demographic characteristics were assessed using t-tests, chi-square tests, or their nonparametric equivalents.

#### Cross-sectional analysis

Spearman correlation matrices were used to examine pairwise correlations of inflammatory markers in LLD patients and controls. Comparisons of 12 biomarkers between PRODE and COGNORM groups were done using multiple linear regression with stepwise adjustment for covariates (age, gender, smoking status, comorbidities). Model assumptions were checked using the Durbin-Watson statistic, VIF, DFBeta coefficients, and Cook’s distances. Heteroscedasticity was addressed the Breusch-Pagan test, and, if necessary, adjusted standard errors.

To investigate the association between depression and high-vs-low levels of plasma inflammation, values of each inflammatory marker were dichotomised at the median. Binary logistic regression was performed with depression status as outcome variable. Odds ratios (ORs) and 95% confidence intervals (CIs) were reported for four models with stepwise adjustment, as described above. Multiple comparisons were controlled using Bonferroni correction, with significance set at p ≤ 0.001 for 48 comparisons.

#### Longitudinal analysis

Predictors of progression to dementia (inflammatory markers and clinical variables) were analyzed using Cox proportional hazards regression with “lowest time to dementia” as time function. Inflammatory marker values were log-transformed to address skewness. To avoid crude violations of the EPV rule and overfitting, we only adjusted for covariates which proved to be significant predictors of progression to dementia in univariate models: age, baseline MMSE, APOE genotype and GMHR score. Hazard ratios (HR) with 95% confidence intervals were calculated. Statistical analysis was conducted using Stata/MP 16.1, images were generated using R ver.2024.04.2.

#### Sensitivity analysis

In the cross-sectional part, sensitivity analysis was performed by excluding controls with a history of depression (n = 8). In addition, we repeated the analysis after excluding control participants whose status at year 3 was different from “cognitively stable” (withdrawn, lost to follow-up or died).

Where 50% or more of cytokine values in either of the cohorts were undetectable or below recommended LLOQ (in IL-17a, IFN-γ, IL-1β, IL-33, and IL-10; see Table S3 in Supplement), a sensitivity analysis was run using logistic regression with depression as outcome - by dichotomising each such cytokine by detectability (detectable/non-detectable).

In the longitudinal part, sensitivity analysis was performed by excluding those who had an MMSE score below 25 post-treatment.

### Missing data and attrition

There was minimal missingness ( < 3.4%) on clinical or demographic data in the cross-sectional analysis. Of the 103 COGNORM participants with cytokine data, 83 (80.58%) were known to have no cognitive decline within three years of baseline assessment; the remaining participants either died (n = 7), withdrew (n = 8) or were lost to follow-up/had insufficient information (n = 5) by Year 3.

In the longitudinal analysis, complete baseline and discharge data were available for 86 and 85% of participants, respectively. Baseline missingness was classified as MAR, and post-treatment data gaps (mostly MMSE and MADRS) were predicted by CGI alone. Complete case analysis is presented.

Outcome data (dementia status) was available for 138 of the initial 152 LLD participants at year 1, and for 129 at year 3 (139 participants had data on at least one follow-up date). Attrition at year 1 was due to death (n = 6), withdrawal from study(n = 6); 2 patients were lost to follow-up. Further dropout by year 3 was mostly due to death (n = 10). Patients who had available data on all four assessments had higher baseline MMSE (p < 0.001) and significantly better improvement (CGI-based) post-treatment (p < 0.01).

### Power calculation

We performed a power calculation to determine the smallest detectable effect size for differences in cytokine concentrations between the PRODE (n = 136) and COGNORM (n = 103) groups, resulting in a Cohen’s d of 0.3674, indicating that the cross-sectional study was powered enough to detect small-to-median effects. The power analysis for Cox regression was conducted for 31 events, an alpha of 0.05, and a continuous baseline predictor. Simulations across hazard ratios (HRs) from 1.5 to 2.0, with 5000 iterations per increment, revealed that approximately 80% power was achieved with an HR of 1.8.

## Results

### Demographic and sample characteristics

#### Cross-sectional analysis: PRODE vs COGNORM

The total sample for the cross-sectional part of the analysis included 239 participants for whom cytokine data was available (n = 136 LLD and n = 103 controls).

Participants in the PRODE cohort were, on average, older than COGNORM participants (p < 0.01), were more likely to be female (p < 0.01) and had fewer years of education (p < 0.001). The full comparison of demographic and clinical characteristics between controls and participants with LLD is presented in Table 1.

**Table 1:** The comparison of demographic and clinical characteristics between patients with LLD (n = 136) and controls (n = 103).

	Depression (N = 136)	Controls (N = 103)	Test statistic; p-value
***Numerical characteristics***			
Age (Mean(SD))	76.10(6.88)	73.55(6.73)	t = −2.87, df(237); p = 0.002
Years of education (Median, IQR)	9 (8–12)	14 (12–17)	z = 8.52; p < 0.001
MADRS (median, IQR)	26(21–31)	1(0–3)	z = 6.71; p < 0.001
MMSE (median, IQR)	27(24–29)	29 (29–30)	z = −13.20; p < 0.001
***Categorical characteristics***			
	N(%)		
Gender (female)	98 (72.06)	53(51.46)	χ2(1)* = 10.69; p = 0.001
Marital status			χ2(3)* = 16.36; p < 0.001
Married	55 (40.44)	66 (64.08)	
Widowed	57 (41.90)	26 (25.24)	
Single	10 (7.35)	1 (0.97)	
Divorced/separated	14 (10.29)	10 (9.71)	
***Physical/mental health history***			
Smoking status (yes)	11(10.68)	27(20.61)	χ2(1)* = 4.18; p = 0.04
Cancer	30 (22.06)	35 (33.98)	χ2(1)* = 4.1; p = 0.04
Diabetes	13 (9.56)	8 (7.77)	χ2(1)* = 0.24; p = 0.63
Hypertension	67 (49.26)	38 (36.89)	χ2(1)* = 3.64; p = 0.06
Cerebrovascular	21 (15.44)	14 (13.59)	χ2(1)* = 0.16; p = 0.69
Head injury	35 (26.52)	10 (9.71)	χ2(1)* = 10.55; p = 0.001
Autoimmune dis.	11 (8.09)	6 (5.83)	χ2(1)* = 0.45; p = 0.5
Heart disease	47 (34.56)	20 (19.42)	χ2(1)* = 6.66; p = 0.01
History of depression	91 (67.41)	8 (7.77)	χ2(1)* = 85.54; p < 0.001

N(PRODE/COGNORM) presented for the full sample and the majority of characteristics;

For some variables, N differs due to missingness, namely: Years of education (128/103); MADRS (134/103); MMSE(131/102); Smoking (131/103); Head injury (132/103)

*MADRS* montgomery-åsberg depression rating scale; *MMSE* mini-mental state examination; *SD* standard deviation; *IQR* interquartile range; t Student’s t-test value; z Mann-Whitney U test statistic; *χ*^*2*^ pearson chi-square; *df d*egrees of freedom;

*<20% expected count less than 5.

#### Longitudinal analysis: PRODE

Of 139 patients in the PRODE cohort with outcome data on at least one follow-up date, 36 (25.9%) progressed to dementia; among those, 12 were diagnosed at year 1, and 24 at year 3. Median time to dementia diagnosis was 38.26 months (min 11.51 - max 42.31).

Mean MADRS score on admission was 26.5(SD 8.41). The majority of patients (48.18%) had a severe depressive episode; 14 (10.07%) had psychotic depression. Fourty-four (31.88%) participants had no prior depressive episodes, 17 (12.32%) had one and 77 (55.80%) had two or more prior episodes. Data on age of onset of first depressive episode was available for 133 patients with follow-up data (of them, 33 dementia cases). Median age at first depression onset was 60 (IQR 40–74). Seventy 70 (52.63%) patients were classified as LOD. MADRS scores both at baseline and at discharge was available for 126 patients (34 dementia cases). The average change in MADRS after discharge was −16.24(SD 9.54); the majority (n = 95; 74.5%) responded to treatment, and 51 patient (39.84%) achieved full remission at discharge. The comparison of clinical and demographic variables between those with and without dementia at follow-up is presented in Table 2 and Table S1 in Supplement.

**Table 2:** The comparison of key demographic and clinical characteristics between patients with (n = 36) and without dementia (n = 103) during follow-up.

		Total sample	Dementia (n = 36)	No Dementia (n = 103)	Test statistic; p-value
**Numerical measures**					
Age (mean, SD)		76.23(6.77)	78.86(7.16)	75.31(6.42)	t = −2.77; p = 0.006
Years of education (median, IQR)		9(8–11)	8(7–10)	9(8–12)	z = 1.35; p = 0.18
BMI (median, IQR)		23.68(21.60–26.22)	24.45 (21.80–27.36)	23.42 (21.47–26.18)	z = −1.05; p = 0.30
Age of first onset of depression (median, IQR)		60(40–74)	65(46–77)	60(38–73.5)	z = −1.15; p = 0.25
MADRS (mean, SD)		26.61(853)	25.17(9.70)	27.13(8.06)	t = 1.19; p = 0.24
MADRS change post-treatment (absolute score change - mean,SD)		16.84(9.54)	13.38(9.41)	18.12 (9.31)	t = −2.53; p = 0.013
MMSE (median, IQR)		27(25–29)	25(22–27)	28(26–29)	z = 3.65; p < 0.01
**Categorical measures**					
**N (valid %)**					**χ²(df); p-value/ Fisher’s exact p-value**
Gender	Female	112 (73.7)	25(69.4)	79(76.7)	χ²(1)* = 0.75; p = 0.39
Marital status	Married	67(39.6)	18(36.7)	47(45.2)	Fisher’s exact = 0.07
	Widowed	71(42.01)	24(49)	36(34.6)	
	Single	13(7.7)	1(2.04)	11(10.6)	
	Divorced/Separated	18(10.7)	6(12.24)	10(9.6)	
Smoking	Current smoker	25 (18.66)	4 (11.11)	21 (21.43)	χ²(1)* = 1.85; p = 0.17
Past history of depression	1 vs 0	104(68.9)	25(69.4)	69(67.7)	χ²(1)* = 0.04; p = 0.84
Late-onset depression (cut-off 60)	LOD vs EOD	70 (52.63)	18 (54.55)	52(52.0)	χ²(1)* = 0.06; p = 0.8
ApoE genotype ε4	ε4 carriership	51(37.5)	17(53.1)	29(31.9)	χ²(1)* = 4.57; p = 0.03
allele					
GMHR (Poor health)	Poor vs good	74(48.68)	22(61.11)	42(40.78)	χ²(1)* = 4.44;p = 0.04
CGI	No improvement vs improvement	37(24.50)	12(33.33)	16(15.69)	χ²(1)* = 5.12;p = 0.02

N(N no dementia/ N dementia) presented for full sample, main demographic characteristics and GMHR;

For comparison of all characteristics, see Table S2 in Supplement.

For some variables, N differs due to missingness, namely: years of education: 131 (96/35); BMI: 122(91/31); age of onset: 133(100/33); MADRS baseline: 136(100/36); MMSE baseline: 132 (97/35); improvement on MADRS: 126(92/34); history of depression: 138(102/36); late-onset depression: 133(100/33); ApoE: 123(91/32); smoking:134(98/36); CGI: 138(102/36).

*BMI* body mass index; *MADRS* montgomery-åsberg depression rating scale; *MMSE* mini-mental state examination; *LOD* late-onset depression; *EOD* early-onset depression; *EODGMHR* general medical health rating; *CGI* clinical global impression; *χ*^*2*^ pearson chi-square; *df* degrees of freedom;

*<20% expected count less than 5.

Cytokine data was available for 123 patients with follow-up data; of them 31 had a diagnosis of dementia at follow-up.

### Plasma inflammation in depressed patients vs controls: cross-sectional analysis

The heatmap illustrating intercorrelations between inflammatory markers for controls and participants with LLD is presented in Fig. S1 in Supplement.

**Fig. 1:**
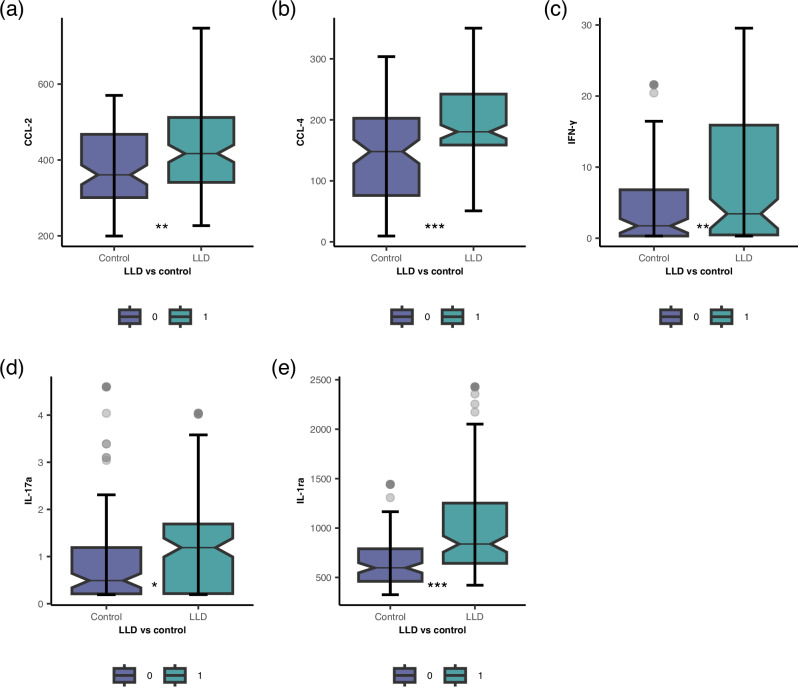
Elevated plasma inflammatory markers in LLD. Boxplots illustrating differences in concentrations of five plasma inflammatory markers between participants with LLD (n = 136) and controls (n = 103). **a** CCL-2, **b** CCL-4, **c** IFN-γ, **d** IL-17a, and **e** IL-1ra were significantly higher in LLD compared to the control group after full adjustment for covariates. For IFN-γ and IL-17a, more than 50% of observations had OOR/below-LLOQ values which were set to 25% of LLOQ (See Table S7 in Supplement). Sensitivity analysis based on detectability levels removed the significance of IFN-γ (see Table S3 in Supplement), suggesting that findings concerning IFN-γ levels should be interpreted with caution. For boxplots illustrating differences in all plasma inflammatory markers, see Fig. S2 in Supplement.. ***p < 0.001; **p < 0.01; *p < 0.05.

Univariate regression analyses revealed significant differences between depressed patients and controls in levels of CCL-2, CCL-4, IFN-γ, IL-17a, IL-1ra, IL-10, IL-6, IL-33 and TNF-α (See Table 3 and Fig. S2 in Supplement). After the first step of adjustment (for age and gender), TNF-α and IL-6 levels were no longer significantly associated with depression. After full adjustment, differences remained significant for CCL-2, CCL-4, IFN-γ, IL-17a, and IL-1ra (see Fig. 1(a–e) and Table 3).

**Table 3:** The results of linear regression models comparing the differences in plasma cytokine levels between LLD (n = 136) and control (n = 103) groups.

	Model 1	Model 2	Model 3	Model 3a	Model 3b	Model 3c	Model 4	R2 (fully adjusted model)
	(β, 95%CI)							
CCL-2	41.94 (11.24–72.64)**	40.73 (8.36–73.10)*	51.20 (17.69–84.70)**	54.69 (20.96–88.42)**	46.59 (12.76–80.43)**	71.78(35.91–107.65)***	52.92(18.92–86.91)**	0.13
CD40L	301.50 (−97.15–700.15)	196.53(−209.60–602.67)	68.98 (−313.82–487.78)	19.69 (−380.56–419.95)	10.41 (−396.51–417.33)	153.42 (−249.94–556.80)	215.61(−195.27–626.50)	0.18
IL-1β	0.11 (−0.04–0.26)	0.10(−0.06–0.27)	0.13(−0.04–0.29)	0.11 (−0.06–0.27)	0.13 (−0.04–0.30)	0.12 (-.06 - .30)	0.10 (−0.72–0.27)	0.04
IL-18	4.61(−13.63–22.85)	11.64 (−6.82–30.10)	6.50 (−12.26–25.25)	8.06 (−11.05–27.18)	7.10 (−11.89–26.10)	9.14(−10.10–28.38)	9.96(−9.55–29.48)	0.14
IL-6	0.62 (0.12–1.11)*	0.33(−0.13–0.78)	0.21 (−0.25–0.67)	0.17 (−0.29–0.62)	0.20 (−0.26–0.66)	0.09 (−0.43–0.61)	0.30(−0.23–0.82)	0.09
CCL-4	57.61 (37.46–78.76)***	43.29 (22.50–64.08)***	35.19 (13.90–56.47)**	37.34 (15.64–59.03)**	34.66(13.11–56.20)**	35.33 (13.35–57.32)**	35.99(14.75–57.23)**	0.19
IFN-γ	4.58 (2.88–6.27***)	4.04(2.25–5.83)***	3.57 (1.59–5.54)***	3.15 (1.17–5.14)**	3.18 (1.16–5.20)**	3.29 (1.21–5.38)**	4.18(2.15–6.20)***	0.15
IL-10	3.01 (0.89–5.14)**	2.13 (−0.16–4.43)	1.71 (−0.79–4.22)	1.70 (−0.75–4.15)	1.70 (−0.71–4.10)	1.69 (-.75–4.12)	2.48(−0.01–4.98)	0.1
IL-17a	0.26 (0.05–0.47)*	0.38(0.17–0.59)***	0.33 (0.09–0.56)**	0.34 (0.11–0.56**)	0.27 (0.04–0.50)*	0.36 (0.10–0.61)**	0.30(0.03–0.56)*	0.07
IL-1ra	253.44 (160.24–346.66)***	234.11(139.24–328.98)***	233.54 (136.20–330.88)***	245.54 (141.56–349.52)***	233.72 (134.49–332.96)***	247.28 (142.63–351.93)***	243.18(129.64–356.72)***	0.11
IL-33	0.46 (0.13–0.79)**	0.46(0.13–0.80)**	0.40 (0.03–0.77)*	0.45 (0.08–0.78)*	0.55(0.17–0.92)**	0.40 (−0.01–0.80)	0.17(−0.25–0.60)	0.04
TNF-α	0.27(−0.21–0.74)	0.25(−0.24–0.74)	0.45 (−0.06–0.96)	0.22 (−0.27–0.72)	0.41 (−0.10–0.92)	0.41 (−0.12–0.93)	0.29(-0.21–0.79)	0.07

*Model 1* unadjusted; *Model 2* adjusted for age and gender; *Model 3* adjusted for age, gender and current smoking.

*Model 3a* adjusted for age, gender, smoking and cardiovascular comorbidity.

*Model 3b* adjusted for age, gender and cancer comorbidity.

*Model 3c* adjusted for age, gender and history of head trauma.

*Model 4* adjusted for age, gender and all comorbidities (cardiovascular, cerebrovascular, cancer, diabetes, autoimmune disorders, history of head trauma).

*Models 3-4* have different (N) due to missing data in the LLD sample:

*Model 3-3b:* N = 234(131/103);

*Model 3c&4*: N = 230(127/103).

***p < 0.001; **p < 0.01; *p < 0.05.

In univariate binary logistic regression using high-vs-low inflammatory marker concentrations as predictors, with account of strict Bonferroni-corrected threshold (p ≤ 0.001), depression was significantly associated with higher levels of CCL-4, IL-1ra, and IFNγ; the associations with CCL-2, IL-33 and TNF-α were significant below the Bonferroni-corrected threshold (See Table S4 in Supplement). In a fully adjusted model, depression was significantly associated with higher levels of IFN-γ and IL-1ra; IL-33, CCL-2, CCL-4 and TNF- α remained significant below the Bonferroni threshold.

### Sensitivity analyses

When participants with a history of depression were excluded from the control group, differences in inflammatory markers remained unchanged.

When we restricted the COGNORM sample to those ascertained as cognitively stable at Year 3, this also did not alter results significantly, although the effect of CCL-2 slightly increased in magnitude (See Table S5 in Supplement).

Sensitivity analysis based on detectability levels was performed for Il-17a, IFN-γ, IL-1β, IL-33, and Il-10. When dichotomised by detectability, none of these markers were significant below the Bonferroni-corrected threshold. IFN-γ was no longer associated with LLD in any of the models. IL-17 lost significance in the fully adjusted model; otherwise, results for IL-1β, IL-33, and Il-10 remained largely unchanged (See Table S6 in Supplement).

### Predictors of progression from LLD to dementia in the PRODE cohort: inflammatory markers and clinical characteristics

In Cox proportional hazards regression, none of the inflammatory markers predicted conversion to dementia either univariately or after adjustment (see Table S7 in Supplement).

Improvement of MADRS score post-treatment was associated with conversion to dementia in the fully adjusted model (HR = 0.95, 95% CI 0.91–0.99, p = 0.014), indicating that an improvement of 1 point on MADRS scale post-treatment was associated with a 5% decreased risk of progression to dementia. Neither response nor remission were significantly predictive of conversion to dementia in univariate models; however, after adjustment, response to treatment appeared significantly associated with lower chance of conversion to dementia (HR 0.45, 95%CI 0.21–0.98, p = 0.043).

Age of onset of first depressive episode and number of past episodes did not emerge as significant predictors (See Table S2 in Supplement).

### Sensitivity analysis

There were 106 patients with MMSE score within the normal range (25 or above) post-treatment; 10 of them had lower MMSE on admission (18–24) but improved with treatment. Twenty-one patients in this group (21.43%) had dementia during follow-up. MADRS improvement no longer emerged as a statistically significant factor, although the trend was maintained (unadjusted HR 0.96, 95%CI 0.92–1.00; p = 0.053). No inflammatory markers were significantly associated with progression to dementia in this group.

## Discussion

The main focus of the present study was to explore the role of a wide range of plasma cytokines and chemokines in LLD. When compared with participants without depression or subsequent cognitive decline, patients with LLD were shown to have elevated concentrations of CCL-2 and CCL-4 and cytokines IL-17a, IL-33, IFN-γ and IL-1ra, as well as IL-33 in most models.

To our knowledge, this is the first study to examine CCL-2 and CCL-4 levels specifically in LLD. Previous meta-analyses in aMDD found significant differences in these chemokines between depressed and control groups [39, 40]. The CCL family is increasingly recognized for its role in neurotransmitter release, blood-brain barrier permeability, and neurogenesis. Overexpression of CCL-2 promotes glial activation and accelerates tau pathology in rodents [41]. In a study on radiation’s impact on neurogenesis, the absence of CCL-2 signaling post-irradiation reduced microglial activation, allowing recovery [42]. CCL-4 is linked to microglial transcription related to Aβ plaque phagocytosis [43] and emerged as a top transcript in rodent models of aging, amyloidosis, and tauopathy [44]. These findings suggest a key role for CCL-2 and CCL-4 in the intersection of affective disorders and neurodegeneration, warranting further research.

Plasma levels of IL-33 were also elevated in LLD in the majority of models, despite losing significance after the most rigorous adjustment. IL-33 has been extensively researched in aMDD; interestingly, in contrast to our findings, a recent meta-analysis pointed to decreased levels of IL-33 in plasma of aMDD patients compared to controls [45]; other studies described its neuroprotective functions and positive effects on cognitive outcomes [46, 47]. At the same time, higher CSF levels of IL-33 were observed in perinatal depression [48]. To our knowledge, this is the first study to address IL-33 concentrations specifically in LLD. A possible explanation for elevated plasma levels of IL-33 in our sample could be the higher permeability of the blood-brain barrier (BBB) in older people [49], resulting in higher peripheral levels of IL-33 in LLD compared to aMDD; or its reactive overexpression in response to acute inflammatory stimulation associated with LLD.

Pro-inflammatory cytokine IL-17a is known to be upregulated in response to stress in rodent models [50], and was shown to be elevated in the serum of patients with aMDD compared to controls [51]. Research in LLD is limited, with just one previous study with negative results [52].

Interleukin-1 receptor antagonist (IL-1ra) is an anti-inflammatory cytokine acting as an inhibitor of IL-1 family cytokines. At least two meta-analyses have shown increased levels of IL-1ra in aMDD compared to controls [9, 53] – despite negative findings in the most recent one [10]. In LLD, one previous study showed elevated plasma IL-1ra which also predicted depression incidence over 6 years of follow-up [54].

Sensitivity analysis erased the significance of IFN-γ, so its elevation should be interpreted cautiously. Meta-analyses on IFN-γ in aMDD have been inconclusive [9, 10, 55], but a study by Piber et al. found it correlated with depressed mood severity in older adults [56].

Importantly, there were substantial differences in the prevalence of physical comorbidities between the two cohorts. While controls were more likely to have a history of cancer and smoking, the LLD group exhibited a much higher prevalence of heart disease and head injury. Although cardiovascular disorders are often associated with elevated cytokines and chemokines [57], correction for cardiovascular comorbidity in our analysis did not alter the results for any markers except IL-6. The stability of our results after adjusting for comorbidities suggests LLD may independently contribute to inflammatory marker elevation.

None of the 12 inflammatory markers we examined significantly predicted progression to dementia. While inflammation is often proposed as a potential shared or mediating mechanism between affective symptoms in older adults and neurodegeneration [8], only three longitudinal studies, to our knowledge, have directly assessed inflammatory markers as predictors of cognitive decline in LLD. Similar to our findings, a study on LLD in patients with diabetes found elevated markers like CRP and IL-6 but no prediction of dementia over a 10.6-year follow-up [58]. In contrast, a community-based study found that the relationship between depressive symptoms (measured by the CESD) and cognitive performance (delayed recall and animal naming) after four years was fully mediated by plasma concentrations of CRP and physical activity [59]. While these findings are not directly comparable to ours due to differences in study design, they highlight CRP as a potential inflammatory marker in LLD and its association with cognitive decline.

Finally, the most recent prospective cohort study identified elevated IL-1β and reduced IL-10 levels as predictors of Alzheimer’s disease in a clinical population of women with LLD [60]. This contrasts with our findings, likely due to differences in study design and sample characteristics. Our cohort consisted primarily of inpatients of both genders, with potentially higher degree of frailty, whereas Pyo et al. applied stricter inclusion criteria, excluding those with “unstable psychiatric features” or baseline MMSE scores below 28. Additionally, the study by Pyo et al. had a had a follow-up of up to 18.53 years, compared to the 3-year follow-up in our study. Although both studies reported identical overall dementia rates (25.76% in Pyo et al. vs. 25.9% in our cohort), patients in our study progressed to dementia (in our case, all-cause dementia, which could also have affected the discrepancy) more rapidly, possibly reflecting the greater severity of their baseline condition and a higher proportion of prodromal dementia manifesting as LLD.

We did not observe any effect of age of onset or number of prior episodes on the risk of progression to dementia – this was confirmed whether we used the cut-off for late onset at 60 or the more inclusive cut-off 50. While it is commonly assumed that LOD is the “culprit” in the depression-dementia association, the bulk of prior research in fact failed to confirm this definitively [61], and one possibility is that it’s the episode in late-life that is responsible for the association rather than onset age [62].

Less improvement during treatment was associated with worse cognitive prognosis: a 1-point improvement in MADRS reduced the risk of dementia by 5%, and response to treatment was associated with a 55% decrease in risk of dementia. One likely explanation for this finding is that at least in a proportion of patients with lack of treatment response, LLD represents an affective manifestation of brain pathology leading to dementia [63]. The role of treatment response has recently been demonstrated by Chan et al. who showed that failure to respond to at least two trials of antidepressants was associated with an almost 2-fold increase in the risk of developing dementia compared to “easy to treat” patients [64]. This points to the value of providing effective and timely treatment for LLD, but also highlights the importance of careful monitoring for LLD patients with poor treatment response. Further prospective studies are needed to identify predictors of prodromal dementia in cases of difficult-to treat LLD and novel therapeutic targets for suspected prodromal dementia manifesting as LLD.

### Strengths and limitations

The strength of the present study lies in the fact that it included a thoroughly phenotyped clinical sample of older depressed inpatients, and analysed a wide array of cytokines.

However, the study also presents certain limitations that warrant consideration. Primarily, although the COGNORM and PRODE cohorts employed analogous metrics for cognitive performance, demographic information, and clinical data, and despite the inflammatory marker analysis being conducted using an identical protocol, these cohorts are essentially separate studies without an intentional effort towards participant matching. This determined the necessity of careful adjustment for demographic and clinical covariates in order to mitigate potential disparities in the samples, which didn’t affect results substantially, as described above.

At the same time, this very distinction also underscores a strength of the study. The COGNORM cohort was intentionally structured to emphasize cognitively intact, non-depressed older people, and the majority of participants included in the baseline analysis (or all participants in sensitivity analysis) were verified to be free of dementia during follow-up. As a result, the cross-sectional analysis offers a rather unique comparison between cases of moderate to severe depression, which could encompass cases of prodromal dementia, and older people known to be both cognitively healthy and cognitively stable over time.

While clinical factors such as age of onset and number of depressive episodes were considered as predictors of dementia, this study did not explore the trajectories of depressive symptoms over time—an important element that might provide further insights into the depression-dementia link. For instance, a recent study of older adults found that the development of dementia over 4.7 years of follow-up was associated with minimal depressive symptoms at baseline but showed a progressively increasing trajectory of symptoms in subsequent annual follow-ups [65]. This suggests that changes in depressive symptoms over time may be key to understanding the depression-dementia relationship.

Another potential limitation is that only plasma cytokine measures were available for both the LLD group and control participants. Whether peripheral inflammation sufficiently reflects CNS inflammation, is debatable. Several studies demonstrated that cytokines efficiently permeate the blood-brain barrier (BBB), suggesting that plasma cytokine concentrations can serve as markers of CNS inflammation [66]; however, studies of cytokine correlations between CSF and plasma in psychiatric patients showed mixed results [48, 67, 68]. Whether plasma measures of inflammation indeed are a valid indicator of neuroinflammation and whether plasma cytokine analysis is a useful tool for exploring the neurobiology of LLD, remains to be determined.

Finally, a notable limitation of the study is the low absolute number of participants with dementia at follow-up, which resulted in limited power for the longitudinal analysis and may explain the lack of observed effects for inflammatory markers. Additionally, our study only included data on all-cause dementia, which may reduce the specificity of our findings. Specific types of dementia, such as Alzheimer’s disease or vascular dementia, may have distinct inflammatory signatures associated with their underlying pathology, which could provide more precise insights into inflammation-dementia links.

## Conclusion

The present investigation demonstrated elevated levels of several plasma inflammatory markers in people with LLD. Nevertheless, none of the markers were able to predict progression from LLD to dementia over the course of three years. Future research should seek to focus more on inflammatory markers specifically in LLD, and examine the potential impact of dysregulated inflammatory markers on neurodegeneration in older people.

## Supplementary information

Supplemental material

## Data Availability

The data that support the findings of this study are not openly available due to reasons of sensitivity and legal restrictions imposed by the registry owners and the ethical committee.
