# Supplementary material for: The role of plasma inflammatory markers in late-life depression and conversion to dementia: a 3-year follow-up study
Source: Mol Psychiatry. 2025 Feb 8;30(7):3029–38. doi: 10.1038/s41380-025-02908-2 (PMC12185312; doi:10.1038/s41380-025-02908-2)
Supplement: Supplementary file 1 — Supplemental material [file 41380_2025_2908_MOESM1_ESM.pdf]

Table S1. The full comparison of demographic characteristics and cognitive test performance between LLD patients with (n=36) and without (n=103) dementia at follow-up

		Total sample**	Dementia (n=36)	No Dementia (n=103)	Test statistic; p-value
Numerical characteristics					
**Age (mean, SD)**		76.23(6.77)	78.86(7.16)	75.31(6.42)	t=-2.77; p=0.006
**Years of education (median, IQR)**		9(8-11)	8(7-10)	9(8-12)	z=1.35; p=0.18
**BMI (median, IQR)**		23.68(21.60-26.22)	24.45 (21.80-27.36)	23.42 (21.47-26.18)	z=-1.05; p=0.30
**Age of first onset of depression (median, IQR)**		60(40-74)	65(46-77)	60(38-73.5)	z=-1.15; p=0.25
**MADRS (mean, SD)**		26.61(853)	25.17(9.70)	27.13(8.06)	t=1.19; p=0.24
**MADRS change post-treatment(absolute score change - mean, SD)**		16.84(9.54)	13.38(9.41)	18.12 (9.31)	t=-2.53; p=0.013
**Neurocognitive tests battery (baseline)**					
**MMSE (median, IQR)**		27(25-29)	25(22-27)	28(26-29)	z=3.65; p<0.001
**COWAT_FAS(median, IQR) baseline**		28(22-36)	34(27-40)	24.5 (19-31)	z = 2.39; p=0.017
**COWAT_CAT(median, IQR) baseline**		26.5 (21-31)	23(18-27)	27.5(21-32.5)	z = 2.86; p=0.004
**CERAD immediate recall (median, IQR) baseline**		16(12.5-19)	14(11-18)	17(13-20)	z = 2.16; p=0.03
**CERAD delayed recall (median, IQR) baseline**		4 (2-6)	4 (3-7)	3(2-5)	z = 2.38; p=0.017
**CERAD recognition (IQR) baseline**		16 (14-20)	18 (14-20)	14 (12-18)	z = 2.00; p=0.047
**COWAT_FAS(median, IQR) post-treatment**		31.5(23-41)	24.5(21-31)	34(24-43)	z = 2.75; p=0.006
**COWAT_CAT(median, IQR) post-treatment**		28 (22-33)	25(20-28)	29.5(23-36)	z = 2.75; p=0.005
**CERAD immediate recall (median, IQR) post-treatment**		19(16-23)	16(14-20)	20(16-23)	z = 2.69; p=0.007
**CERAD delayed recall (median, IQR) post-treatment**		5 (4-8)	3(3-5)	6(4-8)	z = 3.64; p<0.001
**CERAD recognition (IQR) post-treatment**		18 (14-20)	16(12-18)	18(14-20)	z = 2.73; p=0.006
Categorical characteristics					
		**N (valid %)**	**N(valid %)**	**N(valid %)**	**χ^2(df); p-value/Firsher's exact p-value**
**Gender (Female)**		104 (74.82)	25(69.4)	79(76.7)	χ^2(1)* = 0.75; p=0.39
**Marital status**	Married	60 (43.17)	18(36.7)	47(45.2)	Fisher's exact = 0.07
	Widowed	56 (40.29)	24(49)	36(34.6)	
	Single	10 (7.19)	0(0)	10(10.6)	
	Divorced/Separated	13 (9.35)	3(12.24)	10(9.6)	
**Past history of depression**		94 (68.12)	25(69.4)	69(67.7)	χ^2(1)*=0.04; p=0.84
**Late-onset depression (cut-off 60)**		70 (52.63)	18 (54.55)	52(52.0)	χ^2(1)*=0.06; p=0.8
**Late-onset depression (cut-off 50)**		82 (61.65)	22 (66.67)	60(0.6)	χ^2(1)*=0.47;p=0.5
**Number of past depressive episodes**	None	44 (31.88)	11 (30.56)	33 (32.35)	
	1	17 (12.32)	3 (8.33)	14 (13.73)	
	2+	77 (55.80)	22(61.11)	55(53.92)	χ^2(2)*=0.90; p = 0.64
**Severity**	Mild	13 (9.49)	4(11.43)	9(8.82)	Fisher's exact = 0.73
	Moderate	53 (38.69)	14(0.4)	39(38.24)	
	Severe	66 (48.18)	15(42.86)	51(0.5)	
	Other (e.g.unspecified)	5 (3.65)	2(5.71)	3(2.94)	
**ApoE genotype (ε4 carriership)**		46 (37.40)	17(53.13)	29(31.87)	χ^2(1)*=4.57; p = 0.03
**Smoking (current smoker)**		25 (18.66)	4 (11.11)	21 (21.43)	χ^2(1)*=1.85; p=0.17
**Coronary heart disease**		28 (20.14)	11(30.56)	17(16.50)	χ^2(1)*=3.27; p=0.07
**Heart failure**		5 (3.60)	3(8.33)	2(1.94)	Fisher's exact = 0.11
**Hypertension**		69 (49.64)	20(55.56)	13(12.62)	χ^2(1)*=0.68; p=0.41
**Diabetes**		15 (10.79)	2 (5.56)	13(12.62)	χ^2(1)*=1.38;p=0.24
**Cancer**		23 (16.55)	5(13.89)	18(17.48)	χ^2(1)*=0.25;p=0.62
**Cerebrovascular**		21 (15.11)	9(25.0)	12(11.65)	χ^2(1)*=3.71;p=0.05
**Autoimmune**		11 (7.91)	3 (8.33)	8(7.77)	χ^2(1)*=0.01;p=0.91
**Arrythmia**		27 (19.42)	10 (27.78)	17(16.50)	χ^2(1)*=2.17;p=0.14
**COPD**		17 (12.23)	2 (5.56)	15(14.56)	Fisher's exact = 0.23
**Head injury**		39 (28.89)	9 (25.0%)	30 (30.30)	χ^2(1)*=0.36; p=0.55
**GMHR (Poor health)**		64 (46.04)	22(61.11)	42(40.78)	χ^2(1)*=4.44;p=0.04
**CGI (No improvement)**		110 (79.71)	12(33.33)	16(15.69)	χ^2(1)*=5.12;p=0.02
**Response**		95 (75.40)	12(35.29)	18(19.78)	χ^2(1)*=3.27;p=0.07
**Remission**		51 (39.84)	15 (44.12)	36 (38.30)	χ^2(1)*=0.35;p=0.55
**Trail Making Test (Part A) baseline**	1 SD or better	18 (16.22)	1 (4.0)	17 (19.8)	Fisher's exact = 0.025
	1-2 SD	50 (45.05)	11(44.0)	39 (45.4)	
	Worse than 2 SD	35 (31.53)	13 (52.0)	22 (25.6)	
	Unable to complete	8 (7.21)	0(0)	8(9.1)	
**Trail Making Test (Part B) baseline**	1 SD or better	25(22.32)	24(27.9)	1(4)	Fisher's exact = 0.048
	1-2 SD	15(13.39)	12(13.8)	3(12)	
	Worse than 2 SD	11(9.82)	8(9.20)	3(12)	
	Unable to complete	61 (54.46)	43(49.42)	18(72)	
**Trail Making Test (Part A) post-treatment**	1 SD or better	23 (21.10)	21(25.6)	2(7.41)	χ^2(1)*=10.93;p=0.012
	1-2 SD	43 (39.45)	35(42.68)	8(29.63)	
	Worse than 2 SD	34 (31.19)	19(23.17)	15(55.56)	
	Unable to complete	9 (8.26)	7(8.54)	2(7.41)	
**Trail Making Test (Part B) post-treatment**	1 SD or better	25 (22.73)	23(27.71)	2(7.41)	Fisher's exact = 0.045
	1-2 SD	13 (11.82)	10(12.05)	3(11.11)	
	Worse than 2 SD	10 (9.09)	5(6.02)	5(18.52)	
	Unable to complete	62 (56.36)	45(54.22)	17(62.96)	

BMI Body mass index; *MADRS* Montgomery-Åsberg Depression Rating Scale; *MMSE* Mini-Mental State Examination;
COWAT Controlled Oral Word Association Test; *GMHR* General medical health rating; *CGI* Clinical global impression;
SD standard deviation; *IQR* interquartile range; *t* Student's t-test value; *z* Mann-Whitney U test statistic;
χ^2 Pearson Chi-Square; *df* degrees of freedom; *<20% expected count less than 5.
**N(N no dementia/ N dementia) presented for main demographic characteristics, comorbidities and GMHR;
For some variables, N differs due to missingness, namely:
Years of education: 131 (96/35); BMI: 122(91/31); Age of onset: 133(100/33); MADRS baseline: 136(100/36); MMSE baseline: 132 (97/35);
Improvement on MADRS: 126(92/34); CERAD recognition baseline: 112(87/25); CERAD immediate recall baseline: 116(90/26);
CERAD delayed recall baseline: 112(87/25); COWAT category fluency baseline: 114(88/26); COWAT FAS baseline: 115(89/26);
CERAD recognition post-treatment: 102(79/23); CERAD immediate recall post-treatment: 109(83/26);
CERAD delayed recall post-treatment: 105(81/24); COWAT category fluency post-treatment: 104(78/26); COWAT FAS post-treatment: 104(78/26);
History of depression: 138(102/36); Late-onset depression (both thresholds): 133(100/33); Number of episodes: 138(102/36);
Severity: 137(102/36); ApoE: 123(91/32); Smoking:134(98/36); Head injury: 135(99/36); CGI: 138(102/36);
Response: 126(92/34); Remission: 128(94/34); TMT_A baseline 111(86/25); TMT_B baseline 112(87/25);
TMT_A post-treatment 109(82/27); TMT_B post-treatment 110(83/27)

Table S2. Cox proportional hazard regression models assessing the role of phenotypical characteristics of LLD and response to treatment in progression to dementia (N=139; n events = 36)

	**Model 1**	**Model 2**	**Model 3**	**Model 4**	**Model 5**
	HR (95% CI)				
**MADRS baseline**	0.98(0.94 – 1.02)	0.99(0.95-1.03)	0.99(0.95-1.03)	0.98(0.94-1.02)	0.99(0.95-1.04)
**MADRS change post-treatment (1 point improvement)**	**0.96 (0.93 – 0.99)***	0.97(0.93-1.00)	**0.96(0.92-0.99)***	**0.96(0.92-1.00)***	**0.95(0.91-0.99)***
**CGI improvement**	0.5(0.25-1.01)	**0.49(0.24-0.99)***	**0.41(0.20-0.85)***	0.52(0.24-1.12)	**0.42(0.19-0.93)***
**Response**	0.58 (0.28 – 1.17)	0.54(0.26-1.10)	0.52(0.25-1.08)	0.61(0.29-1.29)	**0.45(0.21-0.98)***
**Remission**	1.21(0.61-2.41)	1.20(0.60-2.39)	1.12(0.55-2.27)	1.11(0.54-2.26)	0.72(0.31-1.67)
**History of depression**	1.02 (0.50 -2.09)	0.97(0.47-2.00)	0.99(0.46-2.13)	0.94(0.44-2.04)	0.81(0.35-1.87)
**Age at first depresive episode**	1.01 (0.99-1.03)	1.01(0.99-1.02)	1.00(0.99-1.02)	1.00(0.98-1.02)	1.00(0.99-1.02)
**LOD (cut-off 60)**	1.35 (0.68 – 2.70)	1.05(0.51-2.15)	0.99(0.48-2.02)	0.95(0.45-1.98)	1.09(0.50-2.37)
**LOD (cut-off 50)**	1.21(0.58-2.51)	0.97(0.46-2.07)	1.05(0.49-2.23)	1.00(0.46-2.17)	0.93(0.41-2.13)
**Number of past episodes (1 vs 0)**	0.75(0.21-2.73)	0.61(0.17-2.23)	0.61(0.16-2.39)	0.44(0.11-1.78)	0.33(0.08-1.44)
**Number of past episodes (≥2 vs 0)**	1.07(0.51-2.23)	1.05(0.50-2.19)	1.05(0.48-2.29)	1.06(0.49-2.31)	0.93(0.40-2.14)

Model 1 unadjusted (N total/cases = 139/36); *Model 2* adjusted for age; *Model 3* further adjusted baseline MMSE(N 131/35);

Model 4 further adjusted for GMHR; *Model 5* further adjusted for ApoE status (N 119/31);

LLD Late-life depression; *MADRS* Montgomery-Åsberg Depression Rating Scale;

CGI Clinical global impression; *LOD* Late-onset depression; *HR* hazard ratio; *CI* confidence intervals;

GMHR General Medical Health Rating; *MMSE* Mini-Mental State Examination

*p<0.05

Table S3. Summary statistics for raw values and winsorised values of inflammatory markers in PRODE (n=136) and COGNORM (n=103) cohorts; Also presented are frequencies and percentages of OOR/LLLQ values for each marker for both cohorts.

	Raw values		Winsorised values		N OOR<br>(of those identifiable but below recommended LLOQ)	% OOR<br>(of those identifiable but below recommende d LLOQ)	N OOR<br>(of those identifiable but below recommended LLOQ)	% OOR<br>(of those identifiable but below LLOQ)
**Interleukin**	**PRODE: (N=136)**	**COGNORM (N=103)**	**PRODE: (N=136)**	**COGNORM (N=103)**	**PRODE: (N=136)**		**COGNORM (N=103)**	
**IL-1β**	**Range**: 0.15-7.0<br>**M(SD):** 0.92(1.08)<br>**IQR**: 0.15-14.01	**Range**: 0.15-15.98<br>**M(SD):** 1.18 (2.24)<br>**IQR**: 0.15 - 1.15	**Range**: 0.15-3.09<br>**M(SD):** 0.85 (0.80)<br>**IQR**: 0.15-0.88	**Range**: 0.15-4.52<br>**M(SD):** 0.9(1.1)<br>**IQR:** 0.15-1.15	**98(43)**	**72(31.6)**	**88(33)**	**85.4(32)**
**IL-18**	**Range**: 0.7-1319.24<br>**M(SD):** 196.80 (124.10)<br>**IQR**: 131.47 – 231.91	**Range**: 42.07 - 2037.41<br>**M(SD):** 228.46 (210.66)<br>**IQR**: 137.42 - 259.68	**Range:** 78.8 – 334.91<br>**M(SD):** 189.24 (72.61)<br>**IQR:** 131.47 – 231.91	**Range:** 9.6-217.19<br>**M(SD):** 208.13 (90.35)<br>**IQR:** 137.42 - 259.68	**0**	**0**	**0**	**0**
**IL-6**	**Range**: 0.62-26.11<br>**M(SD):** 3.68(3.97)<br>**IQR**: 1.39-3.79	**Range**: 0.05 - 19.49<br>**M(SD):** 2.74 (2.95)<br>**IQR**: 1.03 - 3.28	**Range:** 0.71 – 12.65<br>**M(SD):** 3.49 (3.25)<br>**IQR**: 1.39 – 3.79	**Range:** 0.52-9.43<br>**M(SD):** 2.60 (2.31)<br>**IQR:**1.03 – 3.28	**0**	**0**	**2(1)**	**1.9(1)**
**CCL-2**	**Range**: 118.20-1113.19<br>**M(SD):** 438.47(158.13)<br>**IQR**: 340.87 - 512.59	**Range**: 77.02 - 996.23<br>**M(SD):** 381.10 (135.79)<br>**IQR**: 299.51 - 465.73	**Range:** 222.92 – 734.97<br>**M(SD):** 434.15 (139.50)<br>**IQR:** 340.87 – 512.59	**Range:** 199.49-570.35<br>**M(SD):** 375.88 (110.17)<br>**IQR:** 299.51-469.65	**0**	**0**	**0**	**0**
**CCL-4**	**Range**: 9.51-1414.50<br>**M(SD):** 202.10 (149.40)<br>**IQR**: 158.66-242.52	**Range**: 9.51 - 466.09<br>**M(SD):** 146.00 (92.87)<br>**IQR**: 299.51 - 469.65	**Range:** 50.76 – 337.95<br>**M(SD):** 188.48 (79.15)<br>**IQR:** 158.66 – 242.52	**Range:** 9.57 – 303.64<br>**M(SD):** 142.55 (82.89)<br>**IQR:** 76.08	**5(1)**	**3.7(0.7)**	**14(0)**	**13.6 (0)**
**CD40L**	**Range**: 2.14-9250.90<br>**M(SD):** 4303.66(1885.34)<br>**IQR**: 2989.43 - 7662.13	**Range**: 7.55 - 23117.96<br>**M(SD):** 4259.46 (2612.32)<br>**IQR**:3056.23 - 5193.55	**Range:** 1216.89 – 7662.13<br>**M(SD):** 4302.98 (1742.08)<br>**IQR:** 2989.43 – 5482.05	**Range:** 1251.55 – 6208.24<br>**M(SD):** 4001.48 (1385.44)<br>**IQR:** 3065.23-5193.55	**2 (1)**	**1.5(0.7)**	**1(0)**	**1(0)**
**IFN-γ**	**Range**: .31 – 41.77<br>**M(SD):** 8.90 (10.32)<br>**IQR**: 0.46 – 16.09	**Range**: 0.19 - 32.21<br>**M(SD):** 4.98(7.42)<br>**IQR**: 0.31 - 6.99	**Range:** 0.31 – 29.55<br>**M(SD):** 8.73 (9.82)<br>**IQR**: 0.46 – 16.09	**Range:** 0.31-21.59<br>**M(SD):** 4.64(6.31)<br>**IQR:**0.31-6.99	**73(19)**	**53.6 (14)**	**72(23)**	**69.9 (22.3)**
**IL-10**	**Range:** 0.76 – 164.56<br>M(SD): 11.30 (23.28)<br>**IQR**: 0.89 – 13.51	**Range**: 0.76 - 109.50<br>M(SD): 7.12(14.11)<br>**IQR**: 0.81 - 7.02	**Range:** 0.81 – 36.84<br>**M(SD):** 8.70 (11.97)<br>**IQR**: 0.89 – 13.51	**Range:** 0.76-29.57<br>**M(SD):** 6.18 (9.59)<br>**IQR:** 0.81-7.02	**95(17)**	**69.9 (12.5)**	**77(8)**	**74.8(7.8)**
**IL-17a**	**Range**: 0.19 – 11.74<br>**M(SD):** 1.34 (1.83)<br>**IQR**: 0.22 - 1.69	**Range**: 0.19 - 19.35<br>**M(SD):** 1.35(2.83)<br>**IQR**: 0.21 - 1.19	**Range:** 0.19 – 4.04<br>**M(SD):** 1.16 (1.13)<br>**IQR**: 0.21 – 1.69	**Range:** 0.19 – 4.6<br>**M(SD):** 0.99 (1.23)<br>**IQR**: 0.21-1.19	**84(23)**	**61.8(16.9)**	**76(32)**	**73.8(31)**
**IL-1ra**	**Range**: 285.87 – 27504.70<br>**M(SD):** 1261.96 (2378)<br>**IQR**: 642.38-1259.11	**Range**: 289.22 - 2400.97<br>**M(SD):** 690.85(355.42)<br>**IQR**: 458.00 - 785.02	**Range:** 421.47 – 2855.72<br>**M(SD):** 1056.30 (623.15)<br>**IQR**: 642.38 – 1259.11	**Range:** 325.73 – 1441.86<br>**M(SD):** 672.18 (287.48)<br>**IQR**: 458 – 794.24	**0**	**0**	**0**	**0**
**IL-33**	**Range**: 0.14-21.19<br>**M(SD):** 2.67 (6.46)<br>**IQR**: 0.59-2.91	**Range**: 0.14 - 32.42<br>**M(SD):** 1.98(3.83)<br>**IQR**: 0.34-2.09	**Range:** 0.14 – 7.32<br>**M(SD):** 2.08 (1.93)<br>**IQR**: 0.59 – 2.91	**Range:** 0.33-6.0<br>**M(SD):** 1.54(1.63)<br>**IQR:**0.34-2.09	**63(50)**	**46,3(36.7)**	**66(38)**	**64.1(36.9)**
**TNF-α**	**Range**: 0.09 - 17.11<br>M(SD): 4.33 (2.62)<br>**IQR**: 3.01-5.37	**Range**: 0.01-13.89<br>M(SD): 3.65(2.16)<br>**IQR**: 2.07-4.50	**Range:** 0.32 – 9.42<br>**M(SD):** 4.24 (2.30)<br>**IQR**: 3.01-5.37	**Range:** 0.73-7.11<br>**M(SD):** 3.56(1.76)<br>**IQR:**2.07-4.5	**9(3)**	**6.6(2.2)**	**4(0)**	**3.9(0)**

M(SD) Mean (Standard deviation); *IQR*: Interquartile range; *OOR* out of range; *LLOQ* lower limit of quantification

Table S4. The associations between low- and high-level inflammation and LLD (n=136)* vs controls (n=103)

	Model 1 (OR [95%CI])	Model 2 (OR [95%CI])	Model 3 (OR [95%CI])	Model 4 (OR [95%CI])
**CCL-2**	1.82[1.11-2.99]*	1.65[0.99- 2.76]	1.84[1.08 - 3.13]*	1.88[1.06 - 3.33]*
**CD40L**	1.27[0.78-2.07]	1.19[0.71 - 2.00]	1.04[0.61 - 1.79]	1.21[0.68- 2.15]
**IL-1β**	1.62[0.99 – 2.65]	1.74[1.03-2.93]*	1.67[0.98-2.87]	1.59[0.90- 2.81]
**IL-18**	1.19[0.73-1.95]	1.53[0.90-2.61]	1.47[0.85-2.53]	1.70[0.94- 3.04]
**IL-6**	1.45[0.89-2.37]	1.26[0.75 - 2.13]	1.13[0.66-1.96]	1.17[0.66 - 2.11]
**CCL-4**	2.70[1.63-4.47]***	2.26[1.34 - 3.83]**	2.15[1.26 - 3.69]**	2.14[1.21 - 3.80]**
**IFN-γ**	3.44[2.05-5.77]***	3.92[2.25 - 6.82]***	3.48[1.98 - 6.12]***	3.77[2.04 - 6.95]***
**IL-10**	1.72[1.04-2.86]*	1.70[1.004-2.88]*	1.77[1.03 - 3.05]*	1.63[0.91 - 2.91]
**IL-17a**	1.52[0.93 -2.49]	1.68[1.00-2.85]*	1.55 [0.90 - 2.65]	1.50[0.84 - 2.68]
**IL-1ra**	3.48 [2.09-5.82]***	3.29[1.93- 5.59]***	3.03[1.76 - 5.22]***	3.09[1.74 - 5.51]***
**IL-33**	2.12[1.29-3.49]**	2.14[1.26 - 3.61]**	1.95[1.14 - 3.34]**	1.80[1.01 - 3.2]*
**TNF-α**	2.07[1.26-3.4]**	1.85[1.10- 3.11]*	1.95[1.14-3.32]*	1.97[1.12 - 3.47]*

Model 1 unadjusted; *Model 2* adjusted for age and gender; *Model 3* adjusted for age, gender and current smoking

Model 4 adjusted for age, gender and all comorbidities (cardiovascular, cerebrovascular, cancer, diabetes, autoimmune disorders, history of head trauma)

LLD late-life depression; *OR* odds ratio; *CI* confidence intervals; *** p≤0.001 (Bonferroni-corrected significance threshold); **p< 0.01; *p<0.05;

Models 3-7 have different (N) due to missing data in the LLD sample: Model 3: N = 234(131/103); Model 4: N= 230(127/103)

Table S5. Sensitivity analysis: The results of linear regression models comparing the differences in plasma cytokine levels between patients LLD (n=136) and control groups, where the control group is restricted to those known to be cognitively stable by Year 3(n=83)

	Model 1	Model 2	Model 3	Model 3a	Model 3b	Model 3c	Model 4	R2(fully adjusted model)
	(β, 95%CI)							
**CCL-2**	**67.39 (33.92 - 100.86)*** **	**54.95(19.48- 90.42)** **	**67.94 (31.82 - 104.06)*** **	**72.61(35.92- 109.31)*** **	**66.46(29.53- 103.39)*** **	**79.87(42.11- 117.63)*** **	**65.03(26.78- 103.28)** **	0.14
**CD40L**	387.69 (-35.22 - 810.59)	315.29(- 132.21 - 762.77)	203.37 (-239.12 - 645.86)	196.60(- 249.77 - 642.98)	214.10(- 237.26 - 665.46)	240.27(- 198.94 - 679.48)	354.99 (- 98.69 - 808.67)	0.18
**IL-1β**	**0.22(0.40- 0.39)***	0.12 (-0.04 - 0.28)	0.12(-0.07 - 0.31)	0.09(-0.09 - 0.28)	0.10(-0.08 - 0.29)	0.10(-0.10- 0.30)	0.091(-0.10 - 0.28)	0.04
**IL-18**	2.23 (-17.59 - 22.05)	10.55 (-9.52 - 30.61)	7.98(-12.30 - 28.26)	11.95 (- 8.41 - 32.31)	11.10(-9.16 - 31.37)	12.85(-7.45 - 33.15)	8.97(-12.58 - 30.52)	0.14
**IL-6**	**0.69(0.14 - 1.23)***	**0.53 (0.02 - 1.04)***	0.17 (-0.35 - 0.69)	0.09(-0.44 - 0.61)	0.28(-0.27 - 0.84)	0.21(-0.31- 0.73)	0.24(-0.36 - 0.85)	0.09
**CCL-4**	**62.94(41.33- 84.56)*** **	**43.01 (20.45 - 65.57)*** **	**38.06(15.15- 60.96)** **	**35.27(11.64-58.89)** **	**34.49 (11.47 - 57.51)** **	**33.77(9.82- 57.72)** **	**34.06(11.34- 56.77)** **	0.2
**IFN-γ**	**5.69(3.83- 7.56)*** **	**5.24(3.44- 7.04)*** **	**3.72(1.60- 5.84)*** **	**3.82(1.61- 6.04)** **	**3.92(1.81 - 6.04)*** **	**3.47(1.16- 5.79)** **	**4.85(2.76- 6.94)*** **	0.19
**IL-10**	**3.25(0.96- 5.53)** **	2.13(-0.26 - 4.51)	1.48(-1.21 - 4.16)	1.62(-1.12 - 4.35)	1.58(-0.97 - 4.13)	1.18(-1.47 - 3.84)	1.84(-0.85 - 4.54)	*0.08*
**IL-17a**	**0.61(0.39- 0.84)*** **	**0.47(0.26- 0.69)*** **	**0.47(0.25- 0.68)*** **	**0.42(0.19- 0.65)*** **	**0.38(0.16- 0.59)** **	**0.45(0.19 - 0.72)** **	**0.33(0.05- 0.60)***	0.09
**IL-1ra**	**272.92(171.28 - 374.47)*** **	**233.91(133.57 - 334.24)*** **	**235.82(128.71 - 432.93)*** **	**227.26(113.92- 340.60)*** **	**222.48(116.0 - 328.96)*** **	**255.53(140.09 - 370.97)*** **	**239.49(114.99 -364.0)*** **	0.11
**IL-33**	**0.64(0.28 - 1.00)*** **	**0.70(0.34- 1.06)*** **	**0.55 (0.17 - 0.93)** **	**0.57(0.21- 0.94)** **	**0.57(0.20- 0.99)** **	**0.56(0.18- 0.94)** **	0.26(-0.19 - 0.71)	0.14
**TNF-α**	**0.71(0.17- 1.25)** **	0.35(-0.2 - 0.9)	0.41(-0.14 - 0.95)	0.27(-0.27- 0.80)	0.37(-0.18 - 0.91)	0.43(-0.12 - 0.99)	0.47(-0.10 - 3.04)	0.08

Model 1 unadjusted; *Model 2* adjusted for age and gender; *Model 3* adjusted for age, gender and current smoking

Model 3a adjusted for age, gender, smoking and cardiovascular comorbidity

Model 3b adjusted for age, gender and cancer comorbidity

Model 3c adjusted for age, gender and history of head trauma

Model 4 adjusted for age, gender and all comorbidities (cardiovascular, cerebrovascular, cancer, diabetes, autoimmune disorders, history of head trauma)

LLD Late-life depression; *CI* confidence intervals; ***p<0.001; **p<0.01; *p<0.05

Models 3-4 have different (N) due to missing data in the LLD sample:

Model 3-3b: N = 234(131/83);

Model 3c&4 : N= 230(127/83)

Table S6. Sensitivity analysis to account for detectability of five factors with over 50% of values outside detection range:

Associations between detectability (cytokines dichotomized as detectable vs non-detectable/below LLOQ) and LLD(n=136) vs controls(n=103)

	IL-1β_detectable	IFN-γ_detectable	IL-10_detectable	IL-17a_detectable	IL-33_detectable
**Model 1 (OR [95%CI])**	1.17[0.54 – 2.55]	0.99[0.54-1.81]	1.48[0.83-2.66]	1.96[1.10 -3.48]*	2.19[1.29-3.71]**
**Model 2 (OR [95%CI])**	1.12[0.50-2.51]	1.09[0.57 - 2.08]	1.53[0.83-2.81]	1.96[1.08-3.56]*	2.21[1.27 - 3.84]**
**Model 3 (OR [95%CI])**	1.12[0.49-2.60]	0.88[0.45 – 1.75]	1.57[0.84 - 2.96]	1.90 [1.03 - 3.53]*	2.03[1.15 - 3.58]*
**Model 4 (OR [95%CI])**	1.05[0.43- 2.59]	0.89[0.43 - 1.83]	1.50[0.77 - 2.92]	1.82[0.95 - 3.50]	2.20[1.19 - 4.08]*

Model 1 unadjusted; *Model 2* adjusted for age and gender; *Model 3* adjusted for age, gender and current smoking

Model 4 adjusted for age, gender and all comorbidities (cardiovascular, cerebrovascular, cancer, diabetes, autoimmune disorders, history of head trauma)

LLOQ Lower Limit of Quantification*; LLD* Late-life depression; *CI* confidence intervals; ***p<0.001; **p<0.01; *p<0.05

Models 3-7 have different (N) due to missing data in the LLD sample:

Model 3: N = 234(131/83);

Model 4 : N= 230(127/83)

Table S7. Plasma inflammatory markers (log transformed values) as predictors of progression to dementia in LLD (N total = 123; N dementia cases = 31); Cox proportional hazards models

	Model 1	Model 2
	**HR (95% CI)**	
**log_IL-1β**	0.87(0.59-1.28)	0.81(0.54-1.23)
**log_CD40L**	0.95(0.46-1.96)	0.76(0.33-1.77)
**log_IL-18**	1.37(0.59-3.18)	1.17(0.47-2.92)
**log_IL-6**	1.07(0.66-1.73)	0.86(0.48-1.52)
**log_CCL-4**	0.78(0.39-1.55)	0.51(0.26-1.01)
**log_IL-1ra**	1.10(0.54-2.21)	0.82(0.37-1.85)
**log_IFN-γ**	0.85(0.68-1.05)	0.86(0.69-1.06)
**log_CCL-2**	0.62 (0.21-1.88)	0.83 (0.50-1.40)
**log_IL-10**	0.98(0.77-1.24)	0.36(0.10-1.34)
**log_IL-17a**	0.94(0.68-1.32)	1.04(0.74-1.46)
**log_IL-33**	0.81(0.59-1.13)	0.73(0.53-1.02)
**log_TNF-α**	0.99(0.64-1.52)	0.87(0.54-1.41)

Model 1 Unadjusted; *Model* 2 (n= 118; n dementia = 30) adjusted for age, baseline MMSE, ApoE genotype and GMHR

LLD Late-life depression; *HR* hazard ratio; *CI* confidence intervals.

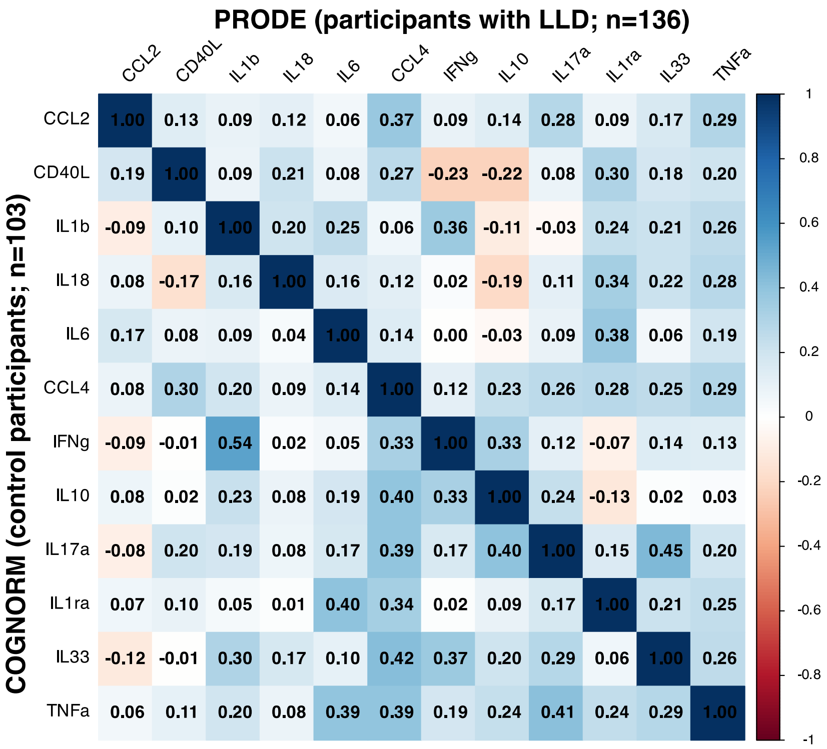

Figure S1. Heatmap displaying Spearman correlation coefficients for pairwise analysis of baseline plasma inflammatory marker levels, shown separately for the control group (COGNORM cohort, n = 103) and the LLD group (PRODE cohort, n = 136).

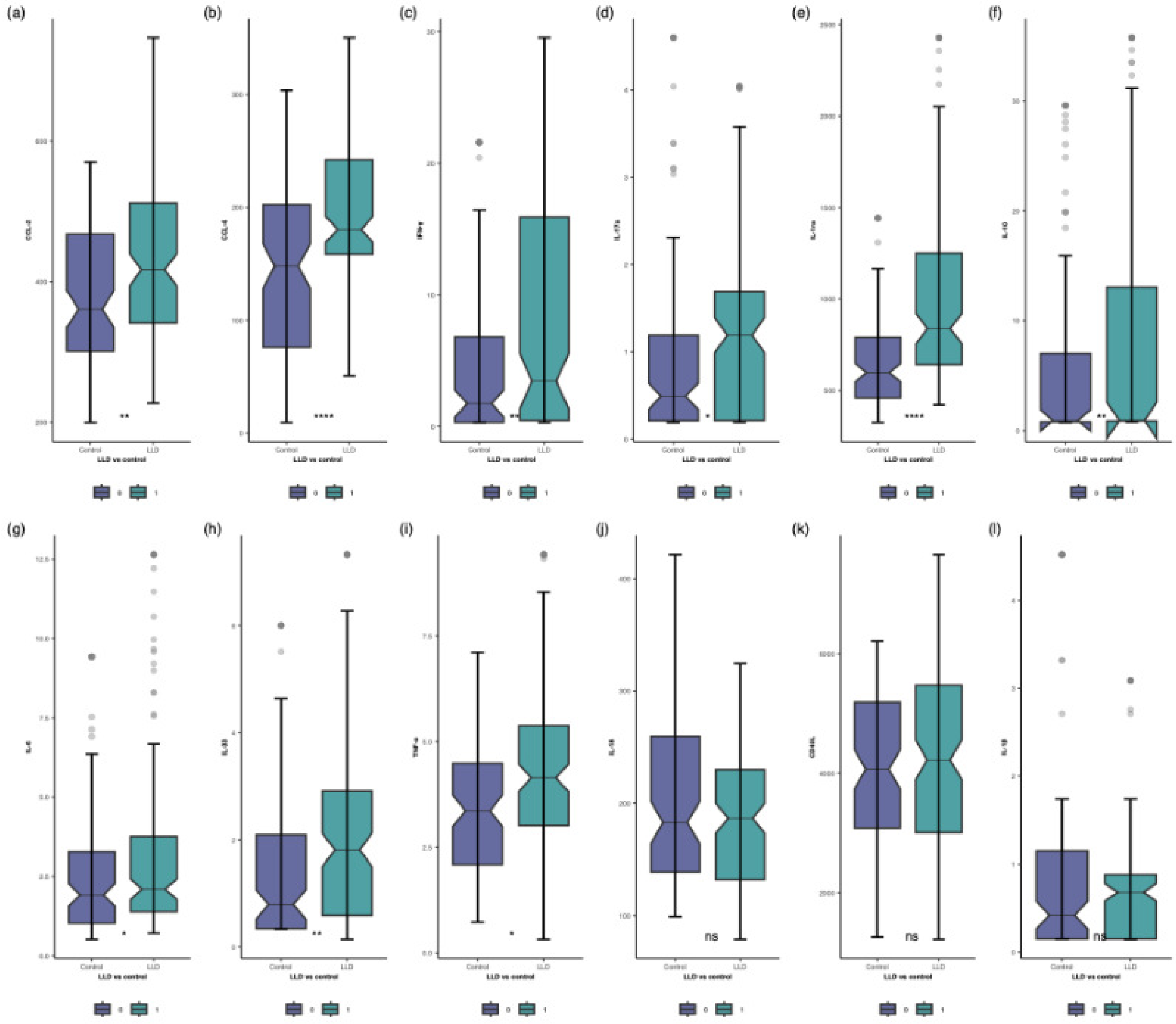

Figure S2. Boxplots illustrating differences in concentrations of all 12 plasma inflammatory markers between participants with LLD (n=136) and controls (n=103): (a)CCL-2; (b) CCL-4; (c) IFN-γ; (d) IL-17a; (e) IL-1ra, (f) IL-10, (g) IL-6, (h) IL-33, (i) TNF-α, (j) IL-18, (k) CD40L, (l) IL-1β. Among these, CCL-2, CCL-4, IFN-γ, IL-17a and IL-1ra emerged as significantly higher in LLD compared to control group after full adjustment for covariates. For IL-17a, IFNγ, IL-1β, IL-33, and IL-10, more than 50% of observations had OOR/below-LLOQ values which were set to 25% of LLOQ (See Table S7 in Supplement). Sensitivity analysis based on detectability levels erased significance for IFN-γ (See Table S3 in Supplement), therefore findings concerning the levels of IFN-γ should be treated with caution.

***$p<0.001$; **$p<0.001$; *$p<0.05$
